# The Role of miR-155 in Nutrition: Modulating Cancer-Associated Inflammation

**DOI:** 10.3390/nu13072245

**Published:** 2021-06-29

**Authors:** Oana Zanoaga, Cornelia Braicu, Paul Chiroi, Nutu Andreea, Nadim Al Hajjar, Simona Mărgărit, Schuyler S. Korban, Ioana Berindan-Neagoe

**Affiliations:** 1 Research Center for Functional Genomics, Biomedicine and Translational Medicine, Iuliu Hatieganu University of Medicine and Pharmacy, 23 Marinescu Street, 40015 Cluj-Napoca, Romania; oana.zanoaga@umfcluj.ro (O.Z.); cornelia.braicu@umfcluj.ro (C.B.); chiroipaul@gmail.com (P.C.); andreeanutu.an@gmail.com (N.A.); ioana.neagoe@umfcluj.ro (I.B.-N.); 2 Department of Surgery, “Octavian Fodor,” Regional Institute of Gastroenterology and Hepatology, 400162 Cluj-Napoca, Romania; na_hajjar@yahoo.com; 3 Department of Surgery, University of Medicine and Pharmacy, 19-21 Croitorilor Street, 400162 Cluj-Napoca, Romania; 4 Department of Anesthesia and Intensive Care I, Iuliu Hatieganu University of Medicine and Pharmacy, 19-21 Croitorilor Street, 400162 Cluj-Napoca, Romania; 5 Prof. Dr. Octavian Fodor Regional Institute of Gastroenterology and Hepatology, 19-21 Croitorilor Street, 400162 Cluj-Napoca, Romania; 6 Department of Natural and Environmental Sciences, University of Illinois at Urbana-Champaign, Urbana, IL 61801, USA; korban@illinois.edu

**Keywords:** miR-155, nutrition, inflammation, cancer

## Abstract

Nutrition plays an important role in overall human health. Although there is no direct evidence supporting the direct involvement of nutrition in curing disease, for some diseases, good nutrition contributes to disease prevention and our overall well-being, including energy level, optimum internal function, and strength of the immune system. Lately, other major, but more silent players are reported to participate in the body’s response to ingested nutrients, as they are involved in different physiological and pathological processes. Furthermore, the genetic profile of an individual is highly critical in regulating these processes and their interactions. In particular, miR-155, a non-coding microRNA, is reported to be highly correlated with such nutritional processes. In fact, miR-155 is involved in the orchestration of various biological processes such as cellular signaling, immune regulation, metabolism, nutritional responses, inflammation, and carcinogenesis. Thus, this review aims to highlight those critical aspects of the influence of dietary components on gene expression, primarily on miR-155 and its role in modulating cancer-associated processes.

## 1. Introduction

Nutrition plays a critical role in the prevention of several serious diseases; however, this role is rather complex, yet it is well orchestrated. To begin with, the genetic profile of an individual is an important player in this game, and in fact, recent genomic breakthroughs are providing a better understanding of the molecular effects of dietary components [1,2,3,4]. Moreover, these are aiding in the development of personalized and targeted enhanced nutrition plans based on both nutritional requirements and the genotype of an individual [1,3]. Recent studies have reported on notable modifications in gene expression resulting from negative effects that nutrients can have on intracellular signaling pathways [1,5,6,7]. Single-nucleotide polymorphisms (SNPs) are the most common types of genetic variations [8]. Due to the fact that miR-155 is a transcriptional product of its host gene (MIR155HG), it has been observed that its expression can be influenced by genetic variations of the MIR155HG gene, as well as of the miR-155 gene [8]. Investigations of variations in miR-155 have been helpful in understanding and delineating observed differences in immunity among individuals [9]. Identified SNPs are reported to be responsible for alterations in levels of mature miR-155 expression and modulations of miR-155-mediated immune responses [9]. Several studies have highlighted the importance of miR-155 and its functional variants in the prevention and prognosis of different diseases, especially cancer. For example, miR-155 and its functional variant rs767649 may contribute to increased risk and poor prognosis of hepatocellular carcinoma [10]. This functional variant, rs767649, in the miR-155 regulation region promotes lung cancer risk and survival [10]. Furthermore, miR-155 can be used as both a diagnostic and prognostic biomarker; i.e., as a potential molecular tool in personalized medicine [11,12,13]. Therefore, to exploit nutrigenomics at an individual level, it is necessary to identify any likely predispositions of the individual for a particular disease. In fact, a family medical history aids in identifying likely risk factors associated with particular pathologies such as hyperlipidemia, obesity, and arterial hypertension [14,15,16].

Various dietary factors, such as sugar, saturated and trans-fatty acids along with some alimentation behaviors such as poor consumption of natural antioxidants, fibers, and omega-3 fatty acids can lead to excessive production of proinflammatory cytokines [17,18]. This is further correlated with reduced production of anti-inflammatory cytokines; thus, resulting in activation of the innate immune system [19,20,21]. Chronic inflammations are correlated with increased risks of developing other related chronic diseases, some of which may lead to mortality, such is the case with diabetes, cardiovascular diseases, and cancer [17,22]. At the molecular level, dietary factors have been noted to have effects on coding and non-coding genes, in particular on key regulatory biological processes [23].

It is known that non-coding RNAs (ncRNAs) are a heterogeneous group of RNA molecules that have either a very low or no potential for protein-coding [24,25,26]. These ncRNAs are reported to be involved in the regulation of gene expression at the transcriptional, translational, and RNA processing levels [24,25,27,28]. The most investigated of ncRNAs are microRNAs (miRNAs), and these are of particular interest as they play major roles in cellular homeostasis and disease. Therefore, the overall goal of this review is to summarize all current knowledge on the influence of dietary components on miRNA gene expression, and in particular on miR-155. This will serve in identifying and elucidating the role of miR-155 in various associated pathologies such as metabolic diseases, obesity, insulin resistance, and cancer.

## 2. MiRNAs and Nutrition

miRNAs are small non-coding RNAs (18–24 nucleotides) and are involved in inhibiting protein translation by binding to 3′-untranslated regions of target mRNAs. This distinct group of nucleic acids serves as important regulators of various cellular processes including differentiation, signaling, and development [26,29,30,31,32]. Dysregulation of specific miRNAs can contribute to different human pathologies such as cardiovascular diseases, metabolic disorders, immune dysfunctions, and cancer [33,34,35]. In their roles in cancer, miRNAs are found to act as both oncogenes, by stimulating tumor growth and metastasis, and tumor suppressor genes, by inhibiting tumor cell proliferation and dissemination, as well as in inducing apoptosis [36,37,38]. Recent studies have demonstrated that miRNAs directly interact with proteins, thereby either regulating gene expression or influencing epigenetic mechanisms [39,40,41].

Remarkably, diet is recognized as one of the most important risk factors. It is associated with many diseases due to phenotypic consequences of adverse dietary habits that have accumulated over the years. For this reason, there is a growing interest in elucidating those mechanisms associated with the involvement of dietary factors in various diseases such as obesity, diabetes, cardiovascular diseases, nonalcoholic fatty liver disease, as well as cancer, as these are attributed to modulations of miRNA expression. Furthermore, these circulating miRNAs have been reported as potential biomarkers of dietary exposure [42]; moreover, their modulation by nutrients could serve as bridges between nutrition and gene expression [42,43]. In addition, it is important to point out that human plasma lipoproteins are correlated with the carrier roles of miRNAs, particularly in the human circulatory system. It is reported that these miRNAs intervene in both physiological and pathological processes due to their high stability; thus, serving as reliable biomarkers for disease status [43,44]. As miRNAs are non-coding RNA transcripts, they play important roles in the regulation of gene expression, as these are retrieved in the bloodstream and are linked to lipoproteins, particularly of high-density lipoproteins (HDL). For example, HDL-miR-92a and miR-486 levels are associated with myocardial infarction and unstable angina, while HDL-miR-223 and miR-24 levels are associated with coronary artery disease [44]. Moreover, the inflammatory transcript miR-155 is retrieved in circulation, and it is bound to HDL [45]. It is reported that miR-155 modulates the SOCS1/STAT3/NF-κB pathway and reduces the production of inflammatory mediators, as it is involved in the oxidation of low-density lipoprotein (LDL) resulting from macrophage inflammatory effects [46].

It has been reported that non-coding genes and hypoxia-inducible factors are targets for selenium in Clear-Cell Renal Cell Carcinoma [47]. Interestingly, reduced expression of miR-155 promotes selenium deficiency-induced apoptosis by TNFRSF1B (tumor necrosis factor receptor superfamily member 1B) in the broiler spleen [48].

## 3. The Role(s) of MiR-155 in Intra- and Intercellular Signaling

miRNAs are critical mediators at the cellular level, and they play important roles in the mediation of intercellular communication [49,50]. In fact, they are deemed key in cell-to-cell signal transduction [51,52].

**Figure 1:**
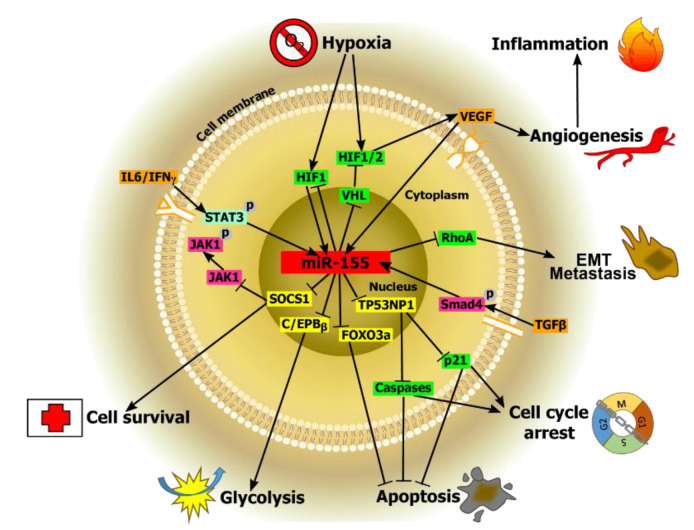
The roles of miR-155 in various biological processes. Hypoxia is a common feature in cancer; thus, different hypoxia-inducible factors (HIF) are found to be responsible for the induction of miR-155 in hypoxic conditions due to associated response elements present in the promoter region of miR-155. Furthermore, miR-155 is highly correlated with inflammatory processes, particularly in lung cancer. This can increase the chances of survival of cancer cells by downregulating the von Hippel–Lindau (VHL) tumor suppressor protein—A protein associated with hypoxia, but this can lead to increased angiogenesis. The transforming growth factor (TGF-) can facilitate metastasis by inducing miR-155 expression via Smad4. By targeting and further reducing the Ras homolog family member A (RhoA) protein, miR-155 can lead to the formation of disrupted tight junctions contributing to epithelial cell plasticity, thereby contributing to invasiveness and migratory processes by TGF- -induced epithelial-mesenchymal transition (EMT). Inhibition/downregulation of miR-155 is reported to induce cell cycle arrest (in the G0/G1 phase) and apoptosis; thus, decreasing cancer cell proliferation in both diffuse large B-cell lymphoma (DLBCL) and clear cell renal carcinoma (ccRC). In a different study, it is found that miR-155 contributes to decreased nasopharyngeal carcinoma cells apoptosis via inhibition of the Caspase 3 activity. Moreover, miR-155 is likely to be involved in glucose metabolism, enhancing insulin sensitivity by negatively regulating the CCAAT/enhancer-binding protein β (C/EBPβ), a negative regulator of the insulin signaling pathway, and thereby further enhancing glycolysis. Finally, miR-155 expression is found to be responsible for downregulation of the suppressor of cytokine signaling 1 (SOCS1) in non-small cell lung cancer (NSCLC), which could further lead to poor survival rates.

As it is known that miR-155 is a microRNA encoded by an miR-155 host gene, miR-155HG (http://www.genenames.org/ (accessed on 13 May 2021)), it plays important roles in different biological processes (Figure 1). These diverse biological processes include inflammation [53,54], immunity [55,56], fibrosis [57,58], autophagy [59,60,61], and carcinogenesis [55,62,63]. It is reported that miR-155 is involved in the control of expression of approximately 250 genes [55,64], and it regulates thiamine, a critical cofactor for some enzymes of the energy metabolism [65]. Recent studies have demonstrated that multiple signaling pathways are involved in the control of the expression of miR-155. For example, regulatory cytokines such as TGF-β can either induce or inhibit miR-155 expression [62,66,67]. Whereas, interferon regulatory factor 3 (IRF3) can suppress neuroinflammation via regulation of miR-155 expression [68]. Moreover, miR-155 is found to regulate IL-17/IL-9-related inflammation in wound healing, thereby demonstrating its potential as a viable therapeutic for decreasing the inflammatory response in wound tissues [69]. In another study, lipopolysaccharide (LPS)-induced miR-155 expression is found to drop in Ets2-deficient mice [70]. Furthermore, miR-155 expression is decreased due to IL-10 inhibition of the transcription factor Ets2. Zheng et al. have identified a nuclear factor (NF)-kappaB (κB) binding site located in the promoter region of the host gene for miR-155, a B-cell integration cluster (BIC) [71]. Coincidently in another study, it is reported that glucocorticoids decrease the expression of miR-155 via inhibition of NF-κB activation. Moreover, miR-155 expression can be regulated by the immune response-associated transcription factor Forkhead box protein P3 (FOXP3), as noted in a recent study of inflammation [72]. In addition, miR-155 regulates levels of expression of SATB1 and ZEB2 in Treg cells [73].

In another thrust, it has been demonstrated that miR-155 regulates a number of genes involved in adipogenesis, as well as those involved in the metabolism of each of carbohydrates and lipids [73]. Adipocyte-derived miR-155 influences diet-induced obesity, and it participates in the polarization of M1 macrophages [74]. In a comparative study of miR-155 expression in male C57BL/6 wild-type (WT) mice and those lacking endogenous miR-155 fed a high-fat diet (HFD) for 6 months, Miller et al. have found that Nr1h3 (liver X receptor alpha [LXRα]) is an miR-155 target gene responsible for the liver phenotype of miR-155(-/-) mice [75]. Interestingly, there is a differential role for miR-155 between genders, wherein miR-155 knockout female mice with a C57BL/6 background, but not male mice, are found to be protected from high-fat diet-induced obesity [76]. This reported finding in knockout female mice is correlated with protection from obesity and with dysfunction of the glucose metabolism. Thus, these findings confirm that there is a decrease in adipose tissue weight in both male and female miR-155 knockout mice, along with an increase of liver steatosis in male miR-155 knockout mice [75,76].

It has been reported that miR-155 is a key regulator of glucose metabolism in breast cancer, and therefore it can positively modulate the energy metabolism via the PIK3R1-PDK/AKT-FOXO3a-cMYC axis [77]. Likewise, the inflammatory response can be regulated due to the coordination of the NF-κB-miR-155 axis with the NF-κB-miR-146a axis [72,78]. Interestingly, miR-155 together with miR-146a are shown to form a unique regulatory network for a specific macrophage inflammatory response via regulation of the NF-κB activity [79]. In another study, miR-155-5p is found to be upregulated, and it is deemed to be responsible for inducing demyelination via upregulation of the common Nogo receptor (NgR), as well as via suppression of the Smad signal transducer protein cascades in male C57BL/6 mice fed a diet supplemented with 0.2% cuprizone [80]. Moreover, in vitro and in vivo studies investigating the relationship between allyl-isothiocyanate and miR-155 have noted a moderate down-regulation of interleukin-1β and miRNA-155 levels, thereby exhibiting a potent anti-inflammatory activity [81].

## 4. The Role of MiR-155 in Regulation of the Immune Response

miRNAs can modulate the host immune homeostasis and immune response through negative regulation of mRNA balance and translation [82]. miR-155 is a multifunctional miRNA, found in cells of the immune system as a key element for the immune response [83]. Moreover, miR-155 expression increases in response to either infection or injury [84,85]. It is reported that miR-155 plays an important role in the activation of different types of immune cells including B-and T-cells, macrophages, and dendritic cells [55,82,83]. Furthermore, miR-155 is proposed as a component of the innate immune response to a variety of inflammatory mediators [72,86]. In fact, the proinflammatory transcription factors activator protein-1 (AP-1) and NF-kB are found to be involved in the regulation of miR-155 expression [87,88,89]. In macrophages, AP-1 can mediate the response to the Toll-like receptor (TLR)-3 and the tumor necrosis factor (TNF)-α [87], as well as of NF-kB to the receptor of lipopolysaccharide (LPS) [90].

Studies with knockout mice have revealed that miR-155 contributes to lung airway remodeling by increasing collagen deposition and inflammatory cell infiltrate [91], thereby decreasing IgG1 antibody production and B-cell memory due to the inability to select for high-affinity plasma B cells [88,92]. Furthermore, the expression of both BIC and miR-155 contributes to the regulation of adaptive immunity through tonsillar germinal center B cells [93]. In addition, the expression of miR-155 in B-cell lines is induced via activation of extracellular-regulated kinase (ERK) and c-jun N-terminal kinase (JNK) pathways. Moreover, chromatin immunoprecipitation analysis has revealed the recruitment of FosB and JunB to the miR-155 promoter and B-cell receptor (BCR) activation [89].

As for T cells, it is found that naive T cells derived from miR-155 knockout mice have increased their likelihood of differentiating into Th2 cells, as well as of producing Th2 cytokines such as IL-4, IL-5, and IL-10 [89,92]. Moreover, in response to antigens, T lymphocytes contribute to lower responses and decreased levels of both IL-2 and interferon [88,91]. Furthermore, IFN-γ is identified in T cells as a second miR-155 target, thus indicating that miR-155 can enhance Th1 differentiation in CD4+ T cells by inhibiting IFN-gamma signaling [94].

## 5. The Role of MiR-155 in Metabolism

miRNAs represent a family of post-transcriptional gene repressors, and they are highly associated with the regulation of gene expression under different conditions, as well as in the control of metabolism [95]. Most studies of immunological and metabolic diseases have focused on the role of miR-155 in these diseases. It is found that miR-155 is expressed in immune cells and adipose tissues; thus, it is suggested that miR-155 is involved in these various diseases, including that of diabetes mellitus [73,96,97].

**Figure 2:**
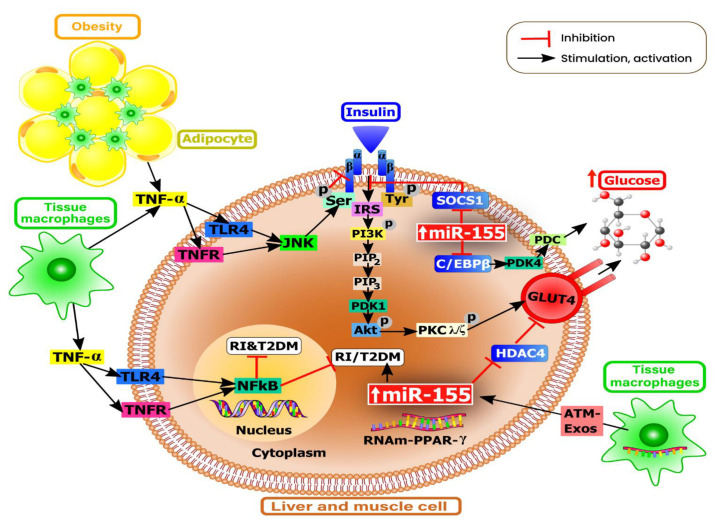
MiR-155 gene regulation in glucose metabolism. MiR-155 positively regulates glucose metabolism via coordinated regulation of multiple genes. Insulin binds to the α-subunit of the insulin receptor (IR). Insulin receptor substrates (IRS), IRS-1 and IRS-2, phosphorylate the p85 regulatory subunit of the phosphoinositide 3-kinase (PI3K). The phosphorylation of PI3K leads to phosphorylation of phosphatidylinositol-3,4-diphosphate (PIP2) into the second messenger phosphatidylinositol-3,4,5-triphosphate (PIP3). Activation of protein kinase (PDK)-1 and serine/threonine-specific protein kinase (Akt) phosphorylates PKCλ/ζ (isoforms of protein kinase C) and vesicles containing the glucose transporter type 4 (GLUT4). Inhibition of the peroxisome proliferator-activated receptor (PPAR)-γ promotes insulin resistance. In tissue macrophages, TNF-α induces insulin resistance via alteration of insulin signaling, thus promoting the development of type 2 diabetes mellitus (T2DM). The JNK promotes insulin resistance through direct phosphorylation of IRS molecules. Moreover, adipose tissue macrophages (ATM) can secrete exosomes (Exos) along with miRNA contents that can reach insulin target cells by promoting insulin resistance and glucose intolerance.

Under normal physiological conditions, miR-155 can maintain standard glucose levels via its involvement in both the regulation of blood glucose homeostasis and insulin sensitivity [98]. However, overexpression of miR-155 in both adipose tissues of the liver and skeletal muscles of transgenic mice is found to promote glycolysis, insulin receptor substrate (IRS)-1 phosphorylation, and the insulin-stimulated serine-threonine kinase AKT (Figure 2). Furthermore, miR-155 is found to mediate the suppression of key negative regulators of insulin signaling such as C/EBPβ, histone deacetylase 4 (HDAC4), and the suppressor of cytokine signaling 1 (SOCS1) [99].

It is worth mentioning that overexpression of miR-155 in liver metabolic processes, demonstrated in Rm155LG/Alb-Cre double-transgenic mice, has resulted in reduced levels of total cholesterol, triglycerides (TG), and free fatty acids both in serum and in hepatic cells, and this is attributed to lower carboxylesterase 3/triacylglycerol hydrolase (Ces3/TGH) [100]. Moreover, Eμ-miR-155 transgenic mice are found to produce higher levels of TNF-α, a key inflammatory cytokine involved in the progression of steatohepatitis in response to LPS treatment [101]. It is important to point out that implication of miR-155 in hepatic metabolism has been first discovered in miR-155 knockout mice fed methionine-choline-deficient (MCD), wherein a series of lipid metabolism-related genes including those encoding for peroxisome proliferator-activated receptor alpha (PPARα), adipose differentiation-related protein (ADRP), and carnitine palmitoyltransferase 1a (CPT1a) are downregulated [102]. In a mouse model using the CRISPR/Cas9 system, it is found that miR-155 regulates high glucose-induced cardiac fibrosis via the TGF-β1–Smad 2 signaling pathway [103]. Moreover, overexpression of miR-155 is induced by high glucose in human renal glomerular endothelial cells, and resulting in increased expression of TNF-α, TGF-β1, and NF-κB [102]. Furthermore, it has been reported that the progression of hyperlipidemia-related diseases such as atherosclerosis, nonalcoholic fatty liver disease (NAFLD), and obesity are mediated by STAT3 and NF-κB due to upregulation of miR-155 expression [104]. Thus, miR-155 may be an important metabolic regulator by playing a role in the mediation of responses to tumor chemotherapy [105].

## 6. The Role of MiR-155 in Inflammation

Inflammation is a complex biological and pathophysiological process that involves responses to infections and injuries; thus, inflammatory mechanisms are associated with many diseases [106,107]. miRNAs have been found to serve as master regulators of inflammation, by modulating inflammatory pathways [106]. In fact, miRNAs are capable of either promoting or suppressing inflammation, depending on the target mRNAs [54,106,108,109].

The role of miR-155 in inflammation has been investigated in various studies. For example, it is found that miR-155 in atherosclerosis is promoted by repressing downstream target genes, such as mitogen-activated protein kinase 10 (MAP3K10), HMG box-transcription protein 1 (HBP1), and B-cell lymphoma 6 (Bcl-6) [110,111]. Moreover, it is reported that miR-155 plays a critical role in retinal neovascularization, wherein downregulation of miR-155 attenuates retinal neovascularization via phosphorylation of effectors in the phosphatidylinositol 3-kinase (PI3K)/protein kinase B (Akt) intracellular signal transduction pathway [112]. Furthermore, miR-155 promotes cholesterol assimilation in THP-1 monocyte-derived macrophages by increasing the expression of scavenger receptors (SRs), LOX-1, CD36, and CD68 [113]. In another study, it is observed that overexpression of miR-155 in macrophages increases the inflammatory response to LPS by targeting the Suppressor of Cytokine Signaling 1 (SOCS-1), thereby disturbing cholesterol efflux from macrophages [114]. In addition, miR-155 has been suggested as an immune-modulatory checkpoint by targeting several molecules involved in the regulation of the immune response, including SMAD2 (smooth-muscle-actin and MAD-related 2) and FOXO3a (Forkhead Box O3) [115,116,117]. Moreover, miR-155–mediated suppression of Bcl6 is critical for acute inflammatory activation of macrophages [110]. Using microarray technology has allowed for the identification of the Toll-like receptor/interleukin-1 (TLR/IL-1) inflammatory pathway, determining its role as a general target of/for miR-155, as well as its implication in the direct control on the level of TAB2, an important signal transduction molecule [86].

In other studies, miR-155-5p is found to be upregulated in murine pancreatic β-cells by hyperlipidemia-associated endotoxemia, thus enhancing glucose metabolism and adaptation of β-cells to obesity-induced insulin resistance (Figure 2) [118]. In an interesting in vivo study on non-alcoholic fatty liver disease (NAFLD), it is observed that upregulation of miR-155 reduces hepatic lipid accumulation by suppressing the liver X receptor alpha (LXRα)-dependent lipogenic signaling pathway. Furthermore, the hexokinase 2(HK2)-modulated glucose metabolism is found to be significantly upregulated in response to overexpression of miR-155, wherein HK2 is found to be an indirect target of miR-155 [119].

## 7. The Role of MiR-155 in Response to Various Dietary Nutrients

Several studies have investigated the roles of miR-155 in response to various nutrient compounds found in the human diet. A listing of these various dietary nutrients and the modulatory effects of miR-155 on biological and molecular targets are presented in Table 1.

Quercetin, a flavonoid compound, widely found in human dietary products such as apples, onions, tea, and red wine is reported to decrease mRNA and protein levels of proinflammatory markers due to a major metabolite, isorhamnetin [120]. For example, it has been found that levels of TNF-α, IL-1β, and IL-6 have significantly dropped in both in vitro-cultured cells and in vivo via either direct down-regulation of miR-155 or by inhibition of the NF-κB pathway [121]. Moreover, overexpression of miR-155 in psoriasis is downregulated by flavonoids and by vitamin D [122].

In T2DM hypertensive medicated patients, resveratrol has been demonstrated to have a favorable immunomodulatory effect via downregulation of the expression of key pro-inflammatory cytokines, as well as of inflammation-related miRNAs, such as miR-155 [123].

Apigenin is another flavonoid abundantly found in many leafy plants and vegetables including chamomile tea, celery, and parsley [124]. It is reported that apigenin reduces LPS-induced miR-155 expression in macrophages via inhibition of NF-κB [115]. Moreover, the expression of both FOXO3a and SMAD2 is increased by apigenin targeting of miR-155 [120]. Thus, TNF-α expression is decreased which, in turn, results in attenuation of inflammatory processes [115]. These findings suggest that dietary apigenin can serve as a low-cost anti-inflammatory nutraceutical.

Curcumin is a natural polyphenol with anti-inflammatory properties due to its effects on the PI3K/AKT signaling pathway [125]. It has been reported that phosphorylation of PI3K, p85a, and AKT levels significantly decrease following curcumin treatment [125]. Moreover, it is demonstrated that the PI3K/AKT pathway is required for the inhibition of miR-155 [126].

In studies with other fruits, it is found that miR-155 is a target of polyphenolic compounds found in pomegranate fruit, which is highly rich in polyphenols along with great bioavailability, wherein miR-155 is downregulated following treatment with these polyphenolic compounds [127]. In addition, it is reported that the downregulation of miR-155 plays a significant role in anti-inflammatory and cytotoxic effects [128].

As various fruits, vegetables, and leafy plants are rich in various vitamins, the effects of these vitamins on the role of miR-155 in alleviating health conditions have received increased interest, and various studies have been undertaken to assess the modulatory effects of miR-155 (Table 1). For example, vitamin C supplements (1250 mg per day) are found to be involved in the regulation of miR-155 expression and are associated with decreases in high-density lipoprotein (HDL) after 8 weeks of supplementation due to reduced levels of reactive oxygen species (ROS) [45]. In this study, miR-155 is significantly downregulated by vitamin C consumption, thereby demonstrating that the circulating miRNA content in HDL can be altered by the dietary intervention [127]. Moreover, it is observed that a high dose of vitamin C elicits antiproliferative and immunomodulatory effects, higher than those of IFN-α, thereby directly decreasing miR-155 expression [125]. In another study, vitamin C-loaded human serum albumin (HSA) nanoparticles are found to downregulate the expression of miR-155, but increased expression of TGF-β1, SMAD1, and SMAD2 [129]. Interestingly, this treatment is found to promote faster healing of wounded tissues in test mice [127].

In vivo studies investigating the influence of 1,25-dihydroxy-vitamin D treatment on macrophages have found that the ligand-bound vitamin D receptor (VDR) signaling downregulates bic gene transcription by blocking NF-κB activation and that miR-155 is positively regulated by TNF-α in human adipocytes [130]. Furthermore, miR-155 deletion attenuates vitamin D suppression of LPS-induced inflammation [131]. In another study focusing on non-small cell lung cancer (NSCLC), it is observed that vitamin D deficiency is correlated with poor prognosis [129]. Furthermore, the capacity of vitamin D to limit inflammation using/with the help of miR-155 has also been demonstrated in adipocytes via the NF-κB signaling pathway [130]. In another thrust, it is reported that human macrophages infected with the dengue virus result in a vitamin D-mediated downregulation of miR-155, and this is attributed to the role of the TLR4/NF-κB/miR-155/SOCS-1 axis in inflammatory response mediation [132].

As polyunsaturated fatty acids (PUFAs) are known to be integrated into cell membranes of various cell types, they interfere with cellular signal transduction by targeting coding and non-coding genes [133]. It has been reported that overexpression of miR-10a, miR-17-3p, miR-125a, miR-155, and miR-181b is observed in response to supplementation of ω-3 PUFA (docosahexaenoic acid) on endothelial cells leaking inflammatory conditions [133]. Under conditions of an inflammatory environment, it is observed that both docosahexaenoic acid (ω-3 PUFA) and arachidonic acid (ω-6 PUFA) activate Toll-like receptor (TLR) signaling and macrophage differentiation [134]. It is reported that these actions are interrelated in the presence of numerous miRNA targets for TLR signaling, including that of miR-155 [133]. In another study, it is reported that miR-155-3p downregulates TLR4 expression in monocytes and macrophages following oleic acid treatment [135].

## 8. MiR-155 in Carcinogenesis and MiRNA-Based Monitoring of Disease Progression

As miR-155 expression is highly observed in various malignancies, various studies have been undertaken to better understand the role of miR-155 in carcinogenesis. A listing of different types of cancers and molecular targets of miR-155 are listed in Table 2.

It has been reported that miR-155 inhibits activated/activation of caspase-3, and induces apoptosis in cancer cells [136,137,138]. Furthermore, miR-155 is observed to act as a negative regulator in *bone morphogenetic protein* (BMP-2)-induced osteogenic differentiation of mesenchymal stem cells (MSCs) [139,140]. Moreover, it increases tumor growth in human hepatocellular carcinoma through Akt phosphorylation by targeting AT-rich interactive domain 2 (ARID2) [141]. In NSCLC, miR-155 induces cell proliferation through inhibition of FOXO1 and increases levels of ROS [142]. Moreover, miR-155 is correlated with poor prognosis as it promotes oral squamous cell carcinoma metastasis [143]. Furthermore, miR-155 induces B-cell lymphoma cell proliferation and inhibits cell apoptosis by targeting nuclear interactors of ARF (alternative reading frame product of the INK4a/ARF locus) and MDM2 (mouse double minute 2) (NIAM) [144].

As miR-155 overexpression is observed in various malignancies including that of lung cancer, it has been found that miR-155 inhibition restores sensitivity of lung cancer to cisplatin via negative regulation of Apaf-1 expression [145]. In another study, miR-155 promotes resistance to tamoxifen, an endocrine therapy for breast cancer patients, by modulating the SOCS6-STAT3 signaling pathway in breast cancer [146]. Furthermore, miR-155 can reverse tamoxifen resistance by activating the suppressor of cytokine signaling 6 (SOCS6), a signal transducer and activator of the transcription 3 pathway in breast cancer [146]. In addition, HK2 is demonstrated to be the first rate-limiting enzyme of glycolysis that is expressed in cancer cells, thereby increasing the glycolytic rate [147,148].

Ectopic overexpression of miR-155 in breast cancer cells promotes upregulation of the key glycolytic enzyme HK2 [149]. It is observed that miR-155 promotes HK2 transcription by activation of STAT3 and by targeting C/EBPβ, an essential protein in regulating genes involved in immune and inflammatory responses [150,151,152].

Interestingly, in vitro and in vivo studies have revealed that a pomegranate extract reduces miR-155 expression while it induces expression of SHIP-1, inhibition of pAkt, and expression of pPI3K [152]. In fact, miR-155 downregulation plays an important role in pathways of immune modulation in adipose-derived mesenchymal stem cells treated with a pomegranate extract (Ad-MSCs) [153]. On the other hand, genistein inhibits breast cancer cell proliferation and down-regulates miR-155 expression thereby inducing caspase-mediated cell death [154]. Furthermore, miR-155 targets FOX03, PTEN, and casein kinase 1α (CK1α), thereby altering β-catenin and p27 expression [155]. Moreover, glycogen synthase kinase-3β (GSK-3β) expression is upregulated in renal carcinoma via inhibition of miR-155, which in turn contributes to inhibition of invasion and cell proliferation, increasing of cell apoptosis, and reducing Wnt/β-catenin activity [156].

The potential role of miR-155 in the regulation of K-RAS has been discovered in chronic myeloid leukemia (CML) patients, wherein higher levels of K-RAS and downregulated expression of miR-155 have been observed [155]. Such a regulation has been proposed as a unique pathway that can be targeted for therapeutic purposes. Interestingly, it has been observed that there is a positive correlation between miR-155 levels and glucose metabolism in 50 triple-negative breast cancer (TNBC) patients with breast tumors via the PIK3R1-PDK/AKT-FOXO3a-cMYC axis [77]. Moreover, hyperlipidemia-associated endotoxemia induces miR-155-5p expression in β-cells, and supports a β-cell phenotype by targeting the transcription factor Mafb that induces repression of IL-6 gene transcription and increasing GLP-1 production [118].

## 9. Conclusions and Perspectives

It is well known that nutrition plays an important role in maintaining human health. Nowadays, nutrigenomics offers wide opportunities in expanding our knowledge of the linkages between nutrition and gene expression towards gaining optimal benefits from our dietary intake.

It is apparent that miRNAs serve as major players in the arena of dietary intake and modulation to enhance human health, as well as in addressing challenges of various pathologies.

Among the diverse groups of miRNAs, miR-155 is a key regulator involved in many biological processes, such as hypoxia, inflammation, angiogenesis, cell cycle arrest, apoptosis, intra- and intercellular signaling, immune regulation, and cell survival, among many others. Moreover, miR-155 is highly correlated with different types of cancers such as colon, pancreatic, breast, and lung cancer by targeting gene expression associated with these malignancies. However, nutrition is found to modulate miR-155 biological outcomes, depending on the molecular target and the dietary compound.

Therefore, nutrigenomics represents an emerging field with promising and positive implications for human health and quality of life. This review emphasizes the important roles of miRNAs in their relationships with the diet for use as therapeutic agents to treat many different diseases. The synergy between nutrition and gene expression, especially of miRNAs, such as that of miR-155, paves the way towards developing new tools that would aid in changing the odds when it comes to untreatable diseases such as cancer. Furthermore, as additional knowledge becomes available, it is expected that there will be a major shift in the food industry, nutritional plans, disease treatment, and medical practice. Nutrition and medicine have always teamed up to maintain human health, but now there is a tremendous opportunity to redefine this relationship by powering through research efforts using new and enhanced nutrigenomic-based tools.

## Figures and Tables

**Table 1:** Molecular targets and biological effects of miR-155 dietary modulation.

Dietary Compound	Study Type	Dose	MiR-155 Expression Level	MiR-155 Targets	Biological Effects	Reference
Allyl-isothiocyanate	In vitro: RAW264.7 macrophages	1–10 μM	↓	↓Nrf2, HO, p65	↓Inflammation	[81]
	In vivo: C57BL/6 mice	15 mg/kg	↓	↓Nrf2, HO, p65	↓Inflammation	[81]
Quercetin	In vitro: RAW264.7 cells	25–100 μM	↓	↓TNF-α	↓Inflammation	[121]
Resveratrol	Clinical study	8 mg/day	↓	↓TNF-α	↓Inflammation	[123]
Curcumin	In vitro/BV2 microglial cells	50 μM	↓	↓PI3K, p85a, and AKT	↓Inflammation	[126]
Apigenin	In vivo/Male C57BL/6J mice	50 mg/kg	↓	↓TNF-α	↓Inflammation	[115]
Pomegranate polyphenolics	In vivo/Female athymic BALB/c nude mice	0.8 mg gallic acid equivalent (GAE)/kg/day	↓	↓NF-kB	↓Inflammation	[128]
Vitamin C	Clinical	1250 mg/day	↓	↓ROS	↓Inflammation ↑Antioxidant activity	[45]
	In vitro/MT-2 cells	100 µg/mL	↓	↑IFN-γ	↑Antiproliferative and immunomodulatory anti-HTLV-1 effects	[125]
	In vivo	20 µg/mL	↓	↑TGF-β1 and SMAD 1,2	↑Wound healing rate	[127]
1,25-dihydroxy-vitamin D	In vivo/mice	20 nM	↓	↓NF-κB	↓Inflammation, innate immunity	[131]
	In vivo/C57BL/6J mice	3000 IU/kg of body weight	↓	↓NF-κB	↓Inflammation	[130]
	In vitro/MDM cells	0.1 nM	↓	↑SOCS-1	↓Inflammation	[24]
PUFAs	In vitro/RAW264.7 cells	15 µmol/L	↓	↓TLR	↓Inflammation	[133]
Oleic acid	In vitro/THP-1 cells	100 μM	↓	↓TLR4	↓ Inflammation	[135]

Abbreviations: PUFAs, polyunsaturated fatty acids; Nrf2, nuclear factor erythroid 2-related factor 2; TNF, tumor necrosis factor; PI3K, phosphoinositide 3-kinases; AKT, protein kinase B; NF-κB, nuclear factor κB; ROS, Reactive oxygen species; IFN-γ, Interferon-gamma; TGF-β1, transforming growth factor beta 1; SOCS1, suppressor of cytokine signaling 1; and TLR4, toll-like receptor 4. ↓-downregulation, ↑-upregulation.

**Table 2:** miR-155 molecular targets in different types of cancer.

Type of Cancer	MiR-155 Expression Levels in Cancer	Targets	Reference
Colon cancer	↑	↓CTHRC1	[157]
Pancreatic cancer	↑	↓SOCS1	[158]
Breast cancer	↑	↓PIK3R1 and FOXO3a	[77]
Chronic lymphocytic leukemia	↑	↓PU.1	[159]
Lung cancer	↑	↓TP53INP1	[160]
	↑	↓PDCD4	[161]
	↑	↓SOCS1, SOCS6, and PTEN	[162]

↑-upregulation, ↓-downregulation.
